# Conformal Coating of Powder by Initiated Chemical Vapor Deposition on Vibrating Substrate

**DOI:** 10.3390/pharmaceutics12090904

**Published:** 2020-09-22

**Authors:** Katrin Unger, Anna Maria Coclite

**Affiliations:** Institute of Solid State Physics, Graz University of Technology, 8010 Graz, Austria; katrin.unger@tugraz.at

**Keywords:** iCVD, initiated chemical vapor deposition, powder coating, conformal coating, vibrating substrate, vibrating powder, thunderstruck, encapsulation of particles

## Abstract

Encapsulation of pharmaceutical powders within thin functional polymer films is a powerful and versatile method to modify drug release properties. Conformal coating over the complete surface of the particle via chemical vapor deposition techniques is a challenging task due to the compromised gas–solid contact. In this study, an initiated chemical vapor deposition reactor was adapted with speakers and vibration of particles was achieved by playing AC/DC’s song “Thunderstruck” to overcome the above-mentioned problem. To show the possibilities of this method, two types of powder of very different particle sizes were chosen, magnesium citrate (3–10 µm, cohesive powder) and aspirin (100–500 µm, good flowability), and coated with poly-ethylene-glycol-di-methacrylate. The release curve of coated magnesium citrate powder was retarded compared to uncoated powder. However, neither changing the thickness coating nor vibrating the powder during the deposition had influence on the release parameters, indicating, that cohesive powders cannot be coated conformally. The release of coated aspirin was as well retarded as compared to uncoated aspirin, especially in the case of the powder that vibrated during deposition. We attribute the enhancement of the retarded release to the formation of a conformal coating on the aspirin powder.

## 1. Introduction

Current coating techniques for encapsulating pharmaceutical powders within polymer films are typically wet spray-based methods. A polymer-solution, -suspension or -emulsion gets atomized into small droplets and floats within a hot carrier gas on a fluidized powder bed. After drying, a thin polymer film with well-defined properties is covering the particles [1]. Even though this technique provides a large variety of coated forms, it is not applicable to all polymers and pharmaceutical powders as toxic solvents may remain in the structure or the pharmaceutics may degenerate under the harsh process conditions. A dry synthesis, such as chemical vapor deposition (CVD) technique, of the polymer directly on the pharmaceutical particles surface are preferential as contaminations with solvents can be neglected [2,3].

Among the possible chemical vapor deposition methods, initiated chemical vapor deposition (iCVD) suits the requirements to coat pharmaceutics as it operates without plasma, works under mild pressure (>100 mTorr) and low substrate temperature (20–60 °C) and is, therefore, also suitable for delicate substrates such as tissue paper, pharmaceutics or even ionic liquids [4,5,6]. iCVD was invented in 2005 by the group of Karen Gleason at the Massachusetts Institute of Technologies and is a vacuum-based free radical polymerization technique [7,8,9]. The initiator species are decomposed by a relatively hot filament (<280 °C) and start the polymerization. At this temperature the monomer species do not degrade, thus their functionality can be retained during the deposition [10,11,12]. Although iCVD seems to be a promising technique to coat pharmaceutical powders or particles, there are only few works on this topic [13,14,15,16]. The standard iCVD reactors fit perfectly the requirements to ensure efficient gas–solid contact on substrates where all surfaces can be reached by the vapors. Indeed, already tubes, overhanging structures or fibers in the sub-micrometer region have been successful conformally coated [16,17,18,19]. Obtaining a conformal coating on powders is more difficult because of the several contact areas among the particles, which prevent the vapors to absorb or diffuse on the whole particle surface. This is especially the case for pharmaceutical powders, compared to other powders such as catalytical powders, which need to be conformally coated over the complete surface area. Since drug release times typically increase with the coating thickness [3]. If a defect—in terms of a non-coated or only little coated area—is present, this point acts as a door that opens easily and lets the drug dissolve through it more quickly than desired. Typical coating techniques for pharmaceutical powders impose movements of the particles to cope with this problem. The previous studies on iCVD particle coatings ensured a good gas–solid contact either by using a 360°-degree rotary cylinder reactor [14] or by manually shaking the powder [15]. The first solution is elegant but needs an elaborate setup. In the second, the deposition process needs to be interrupted several times to open the reactor to manually shake the powder. The drawbacks of this technique are that it does not provide a continuous polymerization throughout the thickness, has statistical non-conformal coating thicknesses and takes a lot of time.

In this study, speakers were introduced in the reactor to shake pharmaceutical powder during the iCVD process. Utilizing sonic vibration during chemical vapor depositions has already proven to enhance the coatings’ conformality on particles [20,21]. Explicitly, a vibration by a non-monotonic frequency is recommended, as played in AC/DC’s song “Thunderstruck”, because it leads to chaotic motion which is beneficial compared to movements caused by monotonic frequencies [22]. The modification of the reactor is easy and can be applied to all common vacuum reactors. For comparison, the powders were also coated on a non-vibrating stage. This configuration, where the gases flow over the particles which do not move, is called flat hearth [3]. For the coating, a polymer of ethylene-glycol-di-methacrylate (p-EGDMA) was chosen. Typically, this monomer is used as a crosslinker in functional polymers to ensure water stability [23,24]. As a homo polymer it forms a strong network that is water-resistant. This monomer was chosen because of its bio-compatibility [25], it is easy to deposit and is a non-functional homo polymer.

Proper model powders for this preliminary study were chosen following the theory of Geldart. In this theory powders can be classified in terms of particle size and density which can be linked to how easy they can be fluidized via gases [26]. Although the theory is about fluidized powder beds and not about in vacuum vibrating powders, the principles, like particle interaction, are also valid in vacuum. The theory classifies powders in four groups. The easiest powders to fluidize fall into groups A and B. Group A gathers powders with small particle size (20–150 µm) and density smaller than 2 g/cm^3^, while group B contains powders composed of particles with a size of 40 µm to 500 µm and a density range between 1–4 g/cm^3^. Group C contains powders which are cohesive mainly due to their small particle size (<20 µm), strong electrostatic forces and Van der Waals attraction. These powders are difficult to fluidize as the attraction forces among the particles dominate over external applied forces. Finally, group D includes the large particles (>500 µm). Powder beds of this group exhibits only little expansion due to fluidization resulting in a poor gas–solid contact.

In this study, two powders were chosen, magnesium citrate and aspirin, which belong to the group C and the group B, respectively. Magnesium citrate is a common dietary supplement and used as food additive to regulate acidity. Further, it is hygroscopic, highly water soluble and has an excellent bioavailability [27,28]. Typically, the powder is white, has a small particle size (<20 µm) and is cohesive with the tendency to agglomerate into clusters. The second powder is aspirin which is a known pain killer and, further, used to treat fever and inflammation [29]. Although it is poorly water soluble it has a good bioavailability due to the good solvability in acid environment, as present in human stomach. The chosen aspirin has a particle size of around 200 µm and falls into group B. The powder can be easily fluidized and gases then have access to the complete particle surface.

## 2. Materials and Methods

### 2.1. Film Preparation

The magnesium citrate powder (by Gatt-Koller—Pharmazeutika, Graz, Austria) and the aspirin powder (Herba Chemosan Apotheker-AG, Graz, Austria) were coated with a polymer of ethylene glycol-dimethacrylate (EGDMA, Aldrich 98%, Vienna, Austria) via iCVD in a custom-built reactor, described elsewhere [30]. The *te*rt-Butyl peroxide (TBPO, Aldrich 98%, Vienna, Austria), which acts as initiator, as well as the EGDMA, were used without further purification and were fed into the reactor with a flowrate of (1.00 ± 0.03) sccm and (0.15 ± 0.02) sccm, respectively. While the TBPO jar was kept at room temperature, the EGDMA monomer was heated to 80 °C. The working pressure during the deposition was 500 mTorr. The temperature of the sample holder was kept at 30 ± 2 °C by a chiller/heater system (Thermo Scientific Accel 500 LC, Thermo Fisher Scientific, Karlsruhe, Germany). The initiator molecules were thermally decomposed by a filament array of nickel-chromium wires (Goodfellow, UK), heated to 270 ± 5 °C. The film thickness was monitored in situ via laser interferometry (He−Ne Laser with λ = 633 nm, Thorlabs GmbH, Dachau, Germany) with a reference silicon wafer sample. The magnesium citrate powder and the aspirin powder were placed in petri dishes.

The magnesium citrate and the aspirin powders were coated simultaneously in the same deposition run. The thickness of the deposition was tracked on a reference silicon wafer which was in an additional petri dish. Afterwards the thickness of the p-EGDMA coating on the silicon wafers was measured via spectroscopic ellipsometry (J. A. Woollam ESM-300) with a spectral region from 370 to 1000 nm under four different incident angles (60°, 65°, 70°, 75°). The data were fitted with a three-layer model containing a silicon and a native silicon (2 nm) layer as substrate and a Cauchy layer, representing the p-EGDMA coating. As previous studies of iCVD depositions on pharmaceutics on flat substrates had shown, a coating thickness of 200 nm is sufficient to provide a retarded release, which is an indication of a closed layer [31,32,33]. Therefore, in this study depositions with coating thicknesses of 100, 200, 300 and 400 nm were performed.

**Figure 1:**
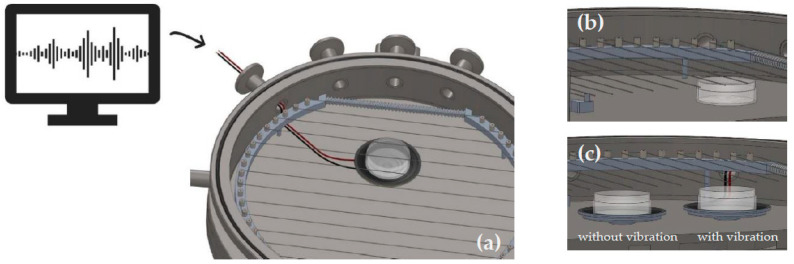
(**a**) overview of an initiated chemical vapor deposition (iCVD) reactor with inserted speaker and petri dish. (**b**) cross section of the setup when performing a flat hearth deposition. (**c**) cross section of the setup when performing depositions with speakers. Both petri dishes are placed on speakers but only one vibrated during the deposition.

In the deposition runs with vibration, the petri dishes with the powders were placed on speakers (Visaton Speaker 2952, 1 W nom, 2 W max, 8 Ω), of which one of them was connected by feed-through from the inside of the reactor to an external amplifier (see Figure 1). These speakers were chosen because of two reasons: First, a petri dish could easily be placed on top of the speaker membrane, fixed by rubber band and, therefore, stayed in good contact with the vibrating area. Secondly, it was flat enough to fit under the filament in the iCVD reactor. During the deposition one speaker was playing “Thunderstruck” by AC/DC (vibration frequency 50–10,000 Hz), while the second one was not vibrating. The second sample should act for direct comparison of powders which were deposited with vibration to that without vibration. The power of the amplifier was increased until the particles of the powder were visibly bouncing. The depositions thickness was 200 nm.

### 2.2. Characterization

Scanning electron microscopy (Jeol JSM-6490LV, Labco GmbH, Pressbaum, Austria) was used to observe the magnesium powder. To diminish electrostatic distortion, a small amount of magnesium powder was smeared on a conductive carbon tape and measured with 20 kV electron beam voltage. Light microscopy (Olympus BX51, Olympus Austria GmbH, Vienna, Austria) was performed to visualize the grain size of the aspirin powder. The particle size distribution was analyzed with the software ImageJ (1.53a). To measure the angle of repose, a photo from the side of a powder pile was taken (KSV Instruments LTD camera 200, KSV Instruments, Helsinki, Finland).

The release tests of the encapsulated magnesium citrate (8 ± 0.2 mg) were performed in demineralized water and the pH of the solution was repeatedly measured with a pH glass electrode (Haoshi H101 pH electrode) connected via a sensor interface to an Arduino. The Arduino powered the glass electrode and was used as a read out terminal. Each 300 ms a pH data point was passed via USB com port to a commercial computer, visualized and stored by a standard application which emulates serial interfaces (Tera Term). The pH values were related to the weight-percentage of the released magnesium citrate through a calibration curve with a 2 mg/mL solution of magnesium citrate in demineralized water. Increments of 0.25 mL were inserted in 150 mL demineralized water and the pH was measured. The pH was related to the inserted weight with a linear fit and, therefore, to the release.

The release test of the encapsulated aspirin was performed with measuring optical absorption spectra (UVVIS Shimadzu 1800, Shimadzu, Korneuburg, Austria) of 3 mg of encapsulated aspirin in 3 mL of demineralized water in time. The aspirin powder was inserted in a quartz cuvette of 1 cm in optical path length and 4 mL in volume and the water was filled into the cuvette with a plastic syringe. In the first hour, a spectrum was taken each 4 min and afterwards each hour in the wavelength region between 190 nm and 350 nm. All spectra were baseline corrected and divided by a reference spectrum of a quartz cuvette with demineralized water. To calibrate the data to link the absorption to the release, in weight percentage of the already dissolved aspirin powder, absorption spectra were taken with solutions of aspirin of known concentrations (1 mg/mL, 0.66 mg/mL, 0.33 mg/mL, 0.166 mg/mL, 0.08 mg/mL, 0.05 mg/mL and 0.027 mg/mL). The peak height of the absorption band of aspirin at 272 nm was linked to the concentration which was used further to calculate the release.

To quantify and compare the time constants of the release from coated and uncoated powders, the release curves were fitted with an exponential model of the form: c (1–e^−bt^). The time τ_c_ and τ_uc_, where the fit of the release of coated powder and the dissolution of uncoated powder exceeded 1/e (36.8%) was determined. The delay factor was calculated with the ratio τ_c_/τ_uc_. A higher delay factor implies a more effective coating that acts as a barrier that slows down the release.

## 3. Results and Discussion

**Figure 2:**
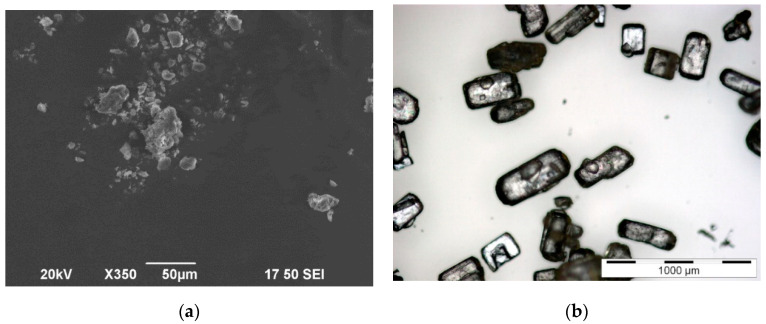
(**a**) Scanning electron microscope image of the magnesium citrate powder. (**b**) Light microscope image of the aspirin powder.

**Figure 3:**
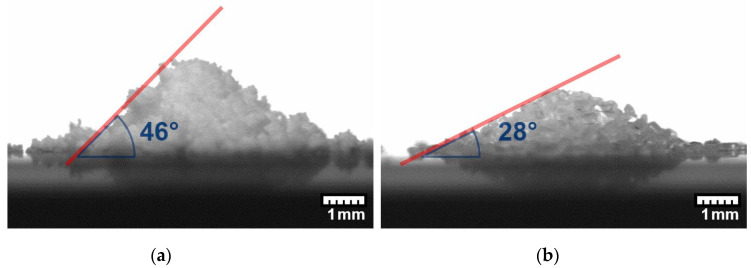
Photo of a powder pile of magnesium citrate (**a**) and aspirin (**b**). Red line marks the angle of repose of the pile.

The grain size and shape of the two powders, magnesium citrate and aspirin, were observed using a microscope. In Figure 2a, the magnesium citrate powder is shown. The particle shape is irregular and the grain size is small with a diameter of (8 ± 9) µm. The particles agglomerate into bigger clusters, which is typical for hygroscopic cohesive powders, such as magnesium citrate. The angle of repose of the magnesium citrate (Figure 3a) was measured to be (46 ± 2)°, suggesting poor flowability [34]. In comparison, aspirin (Figure 3b) has a mean grain size of (200 ± 100) µm. The measured angular of repose of the aspirin (Figure 3b) is (28 ± 2)° suggesting the aspirin particles would have excellent flow properties. The particles are well defined, easily flowing and crystalline. The magnesium citrate and aspirin proved to be of the group C and the group B of the theory of Geldart, respectively [26].

**Figure 4:**
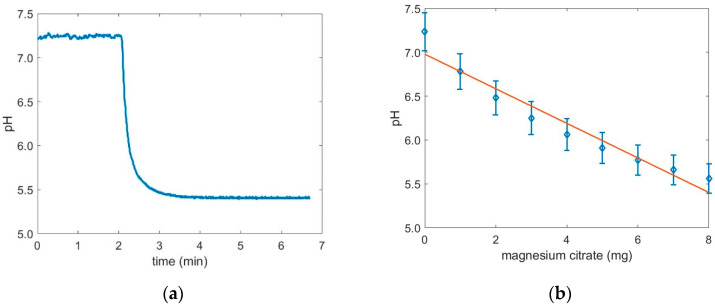
(**a**) Variation of pH in time of dissolving magnesium citrate. (**b**) Calibration curve to link the dissolved magnesium citrate with a pH value.

To measure if the deposition of the magnesium citrate was successful, release tests were performed. The change of pH value in time of a dissolving coated powder in demineralized water was measured. In Figure 4a, the pH value in time of dissolving magnesium citrate without coating is pictured. In the first 2 min there was no powder added to the demineralized water and the pH value was constant at 7.2. After 2 min the powder was added and immediately the pH value dropped from 7.2 to 5.5 within 1 min. This indicates that the magnesium citrate dissolves in water and induces a change in pH, as expected. The calibration curve, which links the pH value with the concentration of dissolved magnesium citrate, is plotted in Figure 4b. The pH value decays with the amount of magnesium citrate. The best fit to the data was found with a linear regression with a coefficient of determination R^2^ of 0.94, while other functions (e.g., exponential decay) lead to larger errors.

**Figure 5:**
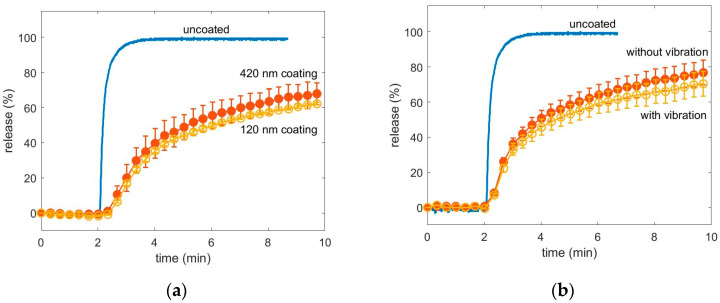
(**a**) Release curve of magnesium citrate coated with a nominal coating thickness of 420 nm and a nominal coating thickness of 120 nm polymer of p-EGDMA. Samples were placed on the reactor bottom as in Figure 1b. (**b**) release curve of magnesium powder coated with and without vibration both with a nominal thickness of 210 nm. Samples were placed on speakers as in Figure 1c.

The release curves of the uncoated magnesium citrate powder, of the one coated with a nominal thickness of 420 nm and with a nominal thickness of 120 nm of p-EGDMA, both in the flat hearth deposition, are compared in Figure 5a. While the uncoated powder dissolved within a minute, the release of the coated powder was retarded. After 10 min about 63% of the magnesium citrate was released. The time, where the dissolution curve of the uncoated powder exceeded 1/e was calculated to be τ_uc_ = (0.22 ± 0.05) min. While the time for magnesium citrate coated with a nominal thickness of 120 nm was τ_c_ = (2.4 ± 0.7) min. The delay factor was estimated to be τ_c_/τ_uc_ = 11.0 ± 0.9 for the powder coated with 100 nm nominal thickness and 9.2 ± 0.4 for the powder coated with 420 nm of nominal thickness. The difference in the release of the powder with nominal 120 nm coating and nominal 420 nm coating is negligibly small which seems unintuitive as thicker coatings should lead to slower release [2].

In Figure 5b, the release curve of magnesium citrate coated with help of a vibrating powder bed with a nominal coating thickness of 210 nm is plotted. Again, the release of a coated powder is retarded compared to a non-coated powder. After 10 min both, the powder which vibrated and which not vibrated during coating, exceeded 67% of release. The delay factor was calculated to be 6.5 ± 0.7 for the powder which was not vibrating and 7.4 ± 0.7 for the vibrating one. Both release curves are within their measurement errors in agreement with each other.

**Figure 6:**
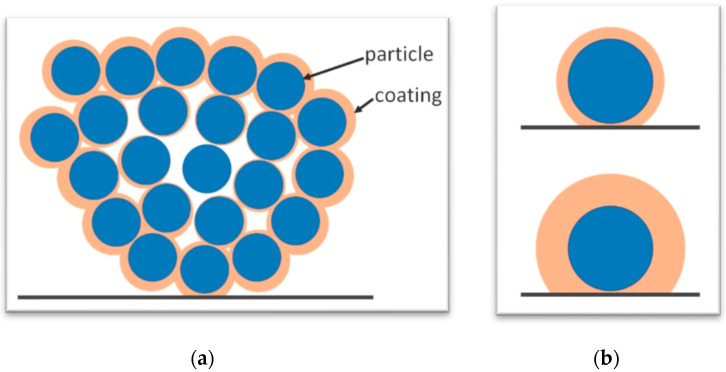
(**a**) Schematic of coating problems in cohesive powder. Due to diffusion limitations and clogging phenomena the inner region of agglomerated clusters is less coated. (**b**) Contact of particles with surfaces are weak points in a flat hearth deposition.

Neither coating thickness nor providing a good gas–powder contact by vibration leads to a change in the release delay. A hypothetical explanation could lie in the cohesion of the magnesium powder. The small particles agglomerate to clusters with a large inner surface area. Due to diffusion limitations or clogging phenomena the coating of the inner area is limited and converge with deposition time. Although the coating of the outer part of the cluster increases, the inner part may be already converged. A schematic of a coated cluster is depicted in Figure 6a. While the surface of the cluster is homogeneously coated, the inner volume has only little coating. With increasing volume, the surface to volume fraction decreases. Therefore, the surface area of larger clusters plays a subordinate role compared to the inner volume of the cluster. Coming back to the measured results this leads to the hypothesis, that the highly cohesive magnesium citrate agglomerates to large clusters, that cannot be disconnect via vibration and, therefore, do not get uniformly coated as such. In this case, when immersing the powder, water penetrates via small voids into the cluster and burst them open. Then the release is mainly driven by the particles, that are in the inner volume of the cluster and neither the coating thickness nor the movement of the powder during deposition has an influence.

**Figure 7:**
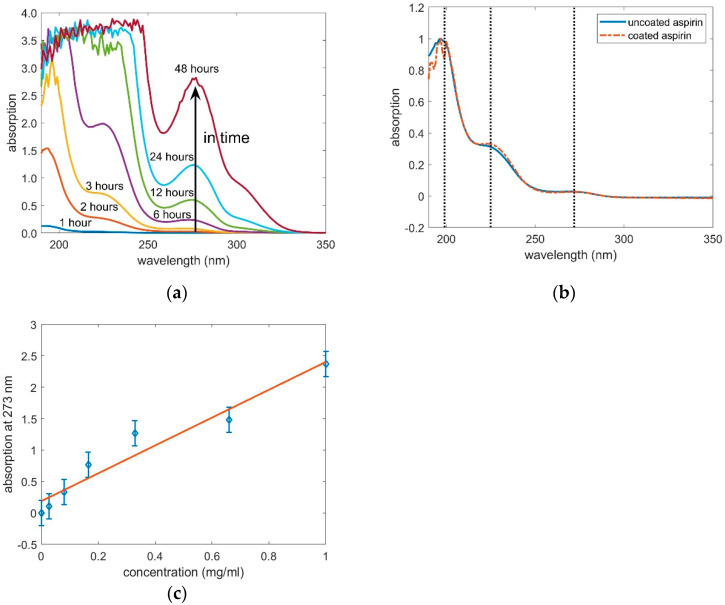
(**a**) Representative absorption spectra of coated aspirin powder. In time the absorption bands of aspirin increase due to the present of dissolved aspirin. (**b**) Scaled absorption spectra of uncoated aspirin dissolved within 10 min and coated aspirin released within 5 h. The absorption bands are alike, indicating a retention of chemical structure during the coating process. (**c**) Calibration curve, which links the peak height of the absorption band at 272 nm to the concentration of aspirin in the water.

**Figure 8:**
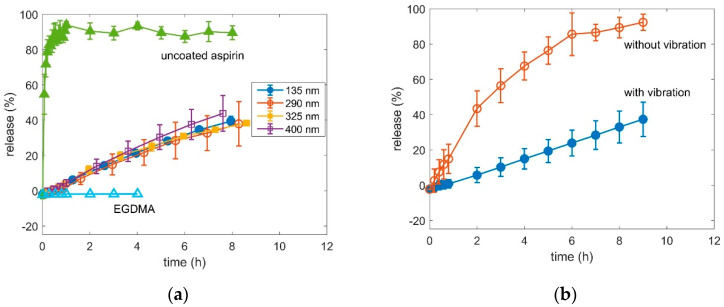
(**a**) Release curve of uncoated aspirin and coated with different thicknesses. Samples were placed on the reactor bottom as in Figure 1b. (**b**) release curve of powder which was vibrating and which was not vibrating during deposition both with a nominal thickness of 200 nm. Samples were placed on speakers as in Figure 1c.

The release of the aspirin was measured with optical absorption measurements. In Figure 7a, a representative of the absorption spectra of coated aspirin in time steps are plotted. With time the typical absorption bands of aspirin at 193 nm, 225 nm and 277 nm rise [35]. Comparing the spectra of uncoated aspirin dissolved within 10 min with coated aspirin released in 5 h, the absorption bands positions and heights are alike (Figure 7b). This indicates, that the release of coated powder is retarded but the chemical structure of aspirin retained during the vibration and the deposition. After approximately 10 h a shoulder at 303 nm appears and starts to increase. This peak is attributed to the formation of salicylic acid. In water, aspirin gradually hydrolyses to acetic and salicylic acids [36]. The height of the absorption band at 277 nm was used in the calibration and is plotted versus the aspirin concentration in Figure 7c. The calibration has approximately a linear trend (linear regression with a R^2^ of 0.94) and was used further to calculate the release, which is the percentage of powder weight that already dissolved. The release of uncoated aspirin is within 30 min at 80% and converges to 85%. The p-EGDMA coating itself has no absorption at 273 nm. The aspirin powder coated with a nominal thickness of 135 nm, 290 nm, 325 nm and 400 nm in the flat hearth deposition is retarded compared to the uncoated aspirin, as it can be seen in Figure 8a. The time where the dissolution of the uncoated powder reached 1/e was calculated with τ_uc_ = (0.13 ± 0.09) h. The time where the release of the powder coated with 135 nm of nominal coating thickness exceeded 1/e was τ_c_ = (7.5 ± 0.9) h. The resulting delay factor was calculated with τ_u_/τ_uc_ = 54 ± 5. For the aspirin powders coated with 290 nm, 325 nm and 400 nm the delay factor were 58 ± 10, 58 ± 4 and 45 ± 10, respectively. The release time of the coated aspirin differs only marginally between different coating thicknesses. The release properties in a flat hearth iCVD deposition cannot be tuned with the deposition thickness. The aspirin does not agglomerate to bigger clusters and the hypothesis of a diffusion limitation to inner particles within the clusters cannot be applied. The typical problem of a flat hearth deposition is the inhomogeneous gas–solid contact. If particles are in contact with the reactor wall or with other particles, the gas cannot reach this surface area. As schematized in Figure 6b, the weak point of the coating is the contact-area from a particle to a surface. Although the upper part of the particle can be coated thicker and thicker, the weak point does not change which would explain why the thickness of the coating has no influence on the release.

The release curves of the aspirin powder which was deposited under vibration is compared to the non-vibrating powder in Figure 8b. For such comparison, the non-vibrating sample was placed at the same height as the vibrating sample, in order to have them at equal distance to the filament and, therefore, at the same temperature. The release of the aspirin which vibrated is much slower than that of non-vibrating aspirin. The delay factors of the release from powder coated without vibration and with vibration was 14 ± 5 and 69 ± 11, respectively. Compared to the delay factor of magnesium citrate coated in the same way, which were 6.5 ± 0.7 and 7.4 ± 0.7 without and with vibration, respectively, the delay of coated aspirin is higher suggesting, that the depositions led to a more effective coating as a release barrier on aspirin then on magnesium citrate. However, while the delay factors of magnesium citrate, coated with and without vibration, differ only within the statistical error, the delay factor of aspirin coated with vibration is significantly higher to that of aspirin coated without vibration.

A vibrating deposition has better gas–solid contact and hypothetically produces a more homogenous coating throughout the complete particle without weak points as it can be suspected under flat hearth conditions, resulting in an enhancement of the retarded release.

## 4. Conclusions

The aim of this study was to show the possibilities and limitations of utilizing speakers to provide vibration of powders during an iCVD deposition. Two different powders, magnesium citrate and aspirin, were coated with a polymer of EGDMA. While in one deposition the thickness of the coating was varied, in another deposition the powders were forced to vibrate with aid of speakers, playing “Thunderstruck” of AC/DC. The release of the coated magnesium citrate is retarded compared to an uncoated magnesium citrate, but neither the coating thickness nor vibration during the deposition changes the release time. This could be due to the fact that magnesium citrate is hygroscopic powder with a small particle size that agglomerates to large clusters. During the coating process the inner particles of a cluster are coated less due to clogging or diffusion phenomena. When the powder is immersed in water the cluster breaks and the inner particles are mainly contributing to the release. Therefore, the release of magnesium citrate coated with different thicknesses and the release of the magnesium citrate which were vibrated and which were not vibrated are the same. The release of the coated aspirin powder retarded as well as compared to the uncoated aspirin. While the coating thickness does not change the release time, the vibration during deposition causes a slower release. During the deposition for the variation of coating thickness, the powder is not moving and each particle touches others or walls. These surfaces are not or only have little accessibility during coating and are weak points when immersing the coated powder in water. Although the other surfaces on each particle are coated with different thicknesses, the amount and size of these weak points is statistically the same within each sample, hence the coating thickness has no influence on the release time. The vibration during coating causes a more retarded release. It seems that the vibration provides a better gas–solid contact which caused a uniform coating. This study demonstrates, that with a small adaption of an iCVD reactor with speakers, certain powders (particles >100 µm, good flowability) can be coated uniformly.
